# Mediterranean Diet Social Network Impact along 11 Years in the Major US Media Outlets: Thematic and Quantitative Analysis Using Twitter

**DOI:** 10.3390/ijerph19020784

**Published:** 2022-01-11

**Authors:** Miguel Angel Alvarez-Mon, Cesar I. Fernandez-Lazaro, Maria Llavero-Valero, Melchor Alvarez-Mon, Samia Mora, Miguel A. Martínez-González, Maira Bes-Rastrollo

**Affiliations:** 1 Department of Psychiatry and Mental Health, Hospital Universitario Infanta Leonor, 28031 Madrid, Spain; 2 Department of Medicine and Medical Specialities, Faculty of Medicine and Health Sciences, University of Alcala, 28801 Alcalá de Henares, Spain; mademons@gmail.com; 3 Department of Preventive Medicine and Public Health, School of Medicine, University of Navarra, 31008 Pamplona, Spain; maria.llavero@salud.madrid.org (M.L.-V.); mamartinez@unav.es (M.A.M.-G.); mbes@unav.es (M.B.-R.); 4 Navarra Institute for Health Research (IdiSNA), 31008 Pamplona, Spain; 5 Department of Endocrinology and Nutrition, Infanta Leonor Hospital, 28031 Madrid, Spain; 6 Centro de Investigación Biomédica en Red de Enfermedades Hepáticas y Digestivas (CIBERehd), Instituto Ramón y Cajal de Investigaciones Sanitarias (IRYCIS), 28034 Madrid, Spain; 7 Internal Medicine and Immune System Diseases-Rheumatology Service, University Hospital Príncipe de Asturias, 28801 Alcalá de Henares, Spain; 8 Center for Lipid Metabolomics, Division of Preventive Medicine, Brigham and Women’s Hospital, Harvard Medical School, Boston, MA 02115, USA; smora@bwh.harvard.edu; 9 Division of Cardiovascular Medicine, Brigham and Women’s Hospital and Harvard Medical School, Boston, MA 02115, USA; 10 Centro de Investigación Biomédica en Red Fisiopatología de la Obesidad y Nutrición (CIBERobn), Institute of Health Carlos III, 28029 Madrid, Spain

**Keywords:** Twitter, social media, Mediterranean diet, public health, health promotion, health communication, mass media, social marketing, journalism

## Abstract

Background: Media outlets influence social attitudes toward health. Thus, it is important that they share contents which promote healthy habits. The Mediterranean diet (MedDiet) is associated with lower cardiovascular disease risk. Analysis of tweets has become a tool for understanding perceptions on health issues. Methods: We investigated tweets posted between January 2009 and December 2019 by 25 major US media outlets about MedDiet and its components as well as the retweets and likes generated. In addition, we measured the sentiment analysis of these tweets and their dissemination. Results: In total, 1608 tweets, 123,363 likes and 48,946 retweets about MedDiet or its components were analyzed. Dairy (inversely weighted in MedDiet scores) accounted for 45.0% of the tweets (723/1608), followed by nuts 19.7% (317/1608). MedDiet, as an overall dietary pattern, generated only 9.8% (157/1608) of the total tweets, while olive oil generated the least number of tweets. Twitter users’ response was quantitatively related to the number of tweets posted by these US media outlets, except for tweets on olive oil and MedDiet. None of the MedDiet components analyzed was more likely to be liked or retweeted than the MedDiet itself. Conclusions: The US media outlets analyzed showed reduced interest in MedDiet as a whole, while Twitter users showed greater interest in the overall dietary pattern than in its particular components.

## 1. Introduction

Over the last decade, access to information and news diffusion have profoundly changed [1]. The internet has modified how people find, communicate, and share information [2]. In fact, the use of the internet as a main source of nutrition information has grown rapidly [3,4]. Gluten-free diet (GFD) has been reported to be the most popular search term among different types of diets in the United States (US) in the years 2005–2015 [5]. In addition, in the US, Google users have shown more interest in Ketogenic or Paleolithic diet than in Mediterranean diet (MedDiet) despite Ketogenic or Paleolithic diet having less scientific foundation [6].

Moreover, social media has become a popular instrument for sharing knowledge [7,8]. Approximately 3.8 billion people around the world are social media users [9]. As a result, most of the media outlets have exponentially increased their dissemination of news through social networks, particularly through Twitter [10]. These messages are a powerful tool because they are capable of influencing public opinion and potentially changing people’s behavior [11,12].

From a public health perspective, the use of Twitter as a source of health information and guidance may be of special relevance [13,14]. This social media enables users to interact with a large number of people, sharing similar interests and concerns about their health and medical conditions [15]. Media outlets are considered to be sensors and drivers of society [16]. Social regard for healthy habits is a relevant factor for the adequate consideration of diseases and for facilitating the general public’s understanding of the importance of lifestyles. Since diet and lifestyles are major determinants of population health, social network contents related to diet and lifestyles may have significant implications for health promotion and public health [17].

In this context, several studies have recently emerged that analyze tweets as a means to understanding trends in public health and evolution of perceptions by the general population on health issues [18]. Several studies have explored the interests and sentiments of the general population regarding certain health problems in Twitter [19,20]. This knowledge is relevant from both a clinical and public health point of view. Previous research has shown that the analysis of social media posts can reveal indicators of the health environment that are associated with area-level mortality and health behaviors, especially in regard to diet and heart disease mortality [21,22]. Yet, there is a lack of data assessing social interest in diet. We attempted to address this gap by examining the distribution of tweets on the topic of the MedDiet or some of its main components in a number of highly recognized and relevant US media outlets. The Mediterranean eating pattern is an overall high-quality food pattern characterized by an abundant. use of olive oil, high consumption of fruits, vegetables, legumes, whole grains, nuts, seeds, and fish, but reduced consumption of dairy products (fermented dairy products are allowed) and limited consumption of red meat, processed meat products and sweets. We selected the MedDiet because of its scientifically proven health benefits, including reduced mortality risk and the prevention of many chronic diseases, particularly cardiovascular diseases (CVD) [23,24,25,26,27,28,29,30,31,32]. We find it interesting and worthwhile to investigate the gap existing between its extensive scientific support and the lack of widespread adherence among the US population. Lastly, we hypothesize that such a discrepancy may be influenced by a lack of diffusion of the positive effects of the Mediterranean diet by the mass media.

The two primary research questions of this study were: (1) To what extent have 25 major US media outlets posted tweets about MedDiet or its components between 2009 and 2019? (2) What type of tweets result in the most engagement (as measured by retweets and likes)?

## 2. Materials and Methods

### 2.1. Collection of Twitter Data

We focused our study on tweets concerning the MedDiet and its components posted by a sample of 25 major US media outlets beginning in January 2009 and concluding in December 2019. We selected a representative sample of different categories of media sites. The selection of sites was carefully conducted to avoid potential bias. We included 11 newspapers (The New York Times, Washington Post, Los Angeles Times, USA Today, The Chicago Tribune, New York Post, Wall Street Journal, New York Daily News, Boston Globe, San Francisco Chronicle, British Daily Mail), 6 broadcast network television or cable news sites (MSNBC, CNN, ABC News, Fox News, CBS News, BBC News), 1 wire service news site (Reuters), 3 hybrid online-only sites (Yahoo news, AOL News, Huffington Post) and 4 pure news aggregators (Google News, The Examiner, Topix and Bing News). These media outlets, which are among the most influential media outlets in the US, though the headquarters of some of them are based in other countries, were selected based on the number of followers on Twitter, as shown by their individual accounts and their social influence during the time of the study [33].

### 2.2. Search Strategy

Our research strategy focused on searching for tweets referring to the MedDiet. We investigated all tweets posted from the Twitter accounts of the previously mentioned media outlets, filtering them according to specific criteria, using the following list of hashtags: #redmeat, #redwine, #softdrinks, #dairy, #nuts, #almonds, #walnut, #hazelnuts, #omega3, #wholegraincereals, #oliveoil, #extravirginoliveoil, #processedmeat, #ultraprocessedfood, #ultraprocessed, #Meddiet and #Mediterraneandiet. We grouped them in eight categories: red meat (#redmeat), red wine (#redwine), soft drinks (#softdrinks), dairy (#dairy), nuts and wholegrains (#nuts, #almonds, #walnut, #hazelnuts, #omega3, #wholegraincereals), olive oil (#oliveoil, #extravirginoliveoil), processed meat (#processedmeat, #ultraprocessedfood, #ultraprocessed) and Mediterranean diet (#Meddiet and #Mediterraneandiet). The inclusion criteria for tweets were: (1) Posted by any of the 25 US media outlets selected for our study; (2) Use of the previously mentioned hashtags; (3) Posted between 1 January 2009 and 31 December 2019; (4) Text in English. An 11-year period (2009–2019) was chosen to align our research with the past decade. We excluded tweets that provided information that was too limited (i.e., tweets consisting mainly of hashtags) or containing only pictures.

### 2.3. Search Tool Used

In this study, we used Twitter Firehose handled by GNIP, which allows access to 100% of all public tweets that match some sort of “search” criteria (query) [34]. In our study, the search criteria were the previously indicated keywords. Tweet Binder, the search engine we employed, uses node.js and PHP language, and is able to analyze tweets in json format (used by GNIP). Next, an individual inspection of all the tweets collected was performed by three members of the research team and resulted in identifying those tweets deemed irrelevant for the purpose of this study. As a result, those tweets that included content not related to nutritional or health-related aspects were excluded, such as those referring to political (e.g., legislation or taxes paid by manufacturers) or economic issues (e.g., sales or transportation logistics). The content of the tweets was then specifically analyzed by three different blinded members of the research team, and the tweets were included when at least two members of the team agreed on the content. This process led to the creation of a more concise database, which yielded a total of 1608 tweets.

### 2.4. Measuring Impact and Sentiment on Twitter

We analyzed the number of retweets and likes generated by each tweet as an indicator of user interest on a given topic. We also measured the potential reach of all analyzed hashtags in order to best assess tendencies in the dissemination of tweets [35]. For the purpose of this study, reach is defined as a numerical value measuring the potential audience of the hashtag. To calculate reach, we added all the followers of each Twitter user (in this case, the 25 US media outlets selected) who participated by posting at least one tweet with any of the hashtags of our study.

In addition, we measured how positive or negative a hashtag was on a scale from 1 (negative) to 100 (positive). We used a modified version of AFINN-165 (a list of English words rated for valence) to calculate a sentiment score for any given tweet. Sentiment analysis tools evaluate all the words contained in each tweet, and each word has its own score that can vary depending on the context [36]. The average score of all the tweets with a certain hashtag determined the overall sentiment score. According to that score, we classified each hashtag in five categories: very negative (0–20), negative (>20–40), neutral (>40–60), positive (>60–80) and very positive (>80–100). Tweet Binder, the search engine we used, provides this analysis.

### 2.5. Ethical Considerations

This study received the approval of the University of Alcala Research Ethics Committee (OE 13_2020) and was compliant with the research ethics principles of the Declaration of Helsinki (7th revision, 2013). However, this study did not directly involve human subjects nor include any interventions but instead used publicly available tweets.

### 2.6. Statistical Analysis

Descriptive statistics were used to summarize tweets, retweets, and likes for each major food component of the MedDiet or the MedDiet as a whole. To adjust likes and retweets for the number of tweets, we calculated the ratio of likes per tweet dividing the number of likes by the number of tweets of each group of food components. Similarly, we applied the same approach for the ratio of retweets per tweet. To analyze the likelihood of a tweet to be liked and retweeted, we calculated odds ratios (OR) and 95% confidence intervals (CI). For that purpose, we first calculated the odds of each food group to be liked and retweeted by dividing the number of likes and retweets by the number of tweets for each group. Then, the odds of each group were divided by the odds of our reference category (soft drinks). Lastly, 95% CI were calculated by applying the following formula: 95% CI = e ^ [ln(OR) ± 1.96 sqrt(1/a + 1/b + 1/c + 1/d)]. The Kruskal–Wallis H test was conducted to determine whether there were statistically significant differences in the number of tweets and retweets by month and year.

## 3. Results

### 3.1. Overall Tweets, Likes and Retweets

A total number of 1608 tweets about the MedDiet and its component food groups were generated by the 25 major media outlets between 2009 and 2019 (Table 1). The number of tweets for MedDiet comprised 9.8% of total tweets (157/1608), while each of the analyzed components of MedDiet followed a heterogeneous pattern of distribution ranging from 1.9% for olive oil (31/1608) to 45% (723/1608) for dairy products. Dairy products, although negatively weighted in most scores assessing adherence to the MedDiet, accounted for almost half of the total number of tweets [37,38,39,40]. The tweets related to nuts accounted for 19.7% of the total (317/1608), and they were followed by MedDiet as a whole and red wine. Additionally, 127 out of the 1608 tweets (7.9%) were related to red meat (also adversely considered in MedDiet scores). Less than 10% of the analyzed tweets (129/1608) referred to the three other components of the Mediterranean diet included in our study. Of note, olive oil, the hallmark of the MedDiet, only accounted for 31 out of the 1608 generated tweets (1.9%) [41].

Next, we investigated the impact of tweets among social media followers by analyzing the responses based on the number of retweets and likes. In total, 48,946 retweets and 123,363 likes were generated (Table 1). We observed a correlation between the number of tweets referring to each component and the number of subsequent retweets and likes generated. Taking into account the number of tweets, the MedDiet as a whole showed the highest number of likes and retweets per tweet (Table 1).

### 3.2. Potential Reach and Sentiment Analysis

**Figure 1:**
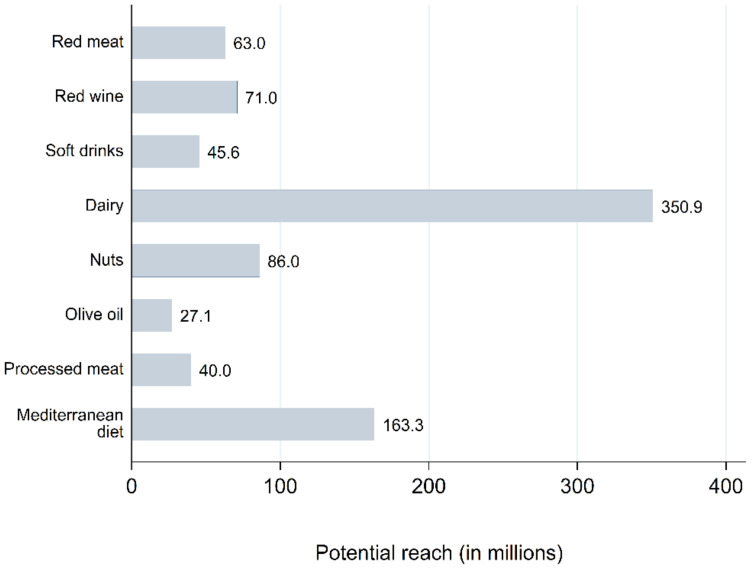
Potential reach (potential audience) of each food group analyzed. Potential reach is defined as a numerical value measuring the potential audience of the hashtag.

Dairy products were the component with the highest potential reach throughout the 11 years of the study (350.9 million, Figure 1). MedDiet as a whole had the second-highest reach (163.3 million), which is less than half of the value obtained by dairy products. The rest of the components (red meat, red wine, soft drinks, nuts, olive oil and processed meat) each obtained a much lower potential reach, being below 90 million for each of them, with the least reach for olive oil at 27.1 million. There were clear differences between dairy products and the rest of the components. The potential reach obtained by dairy products, the component with the greatest reach, was almost 13 times higher than the reach obtained by olive oil, the component with least reach (350.9 and 27.1 million, respectively) despite being the most prominent element in a proper definition of the MedDiet. Indeed, the sum of the reach for these six components (332.7) was lower than that obtained by dairy products (350.9).

**Figure 2:**
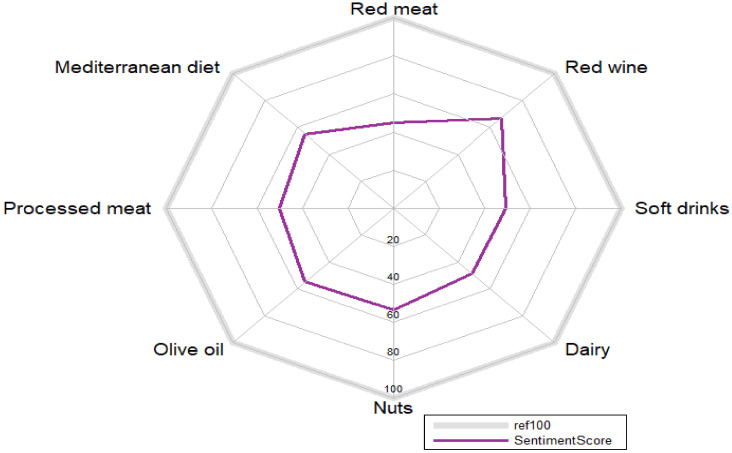
Sentiment analysis of each food group analyzed. The average score obtained by all the tweets posted with a certain hashtag determines the overall score of each food group.

Regarding the sentiment analyses (Figure 2), all components analyzed obtained a score between 40 and 60, which is considered neutral, except for red wine that scored above 60. None of the hashtags analyzed obtained a negative score.

### 3.3. Probability of Being Liked or Retweeted

**Figure 3:**
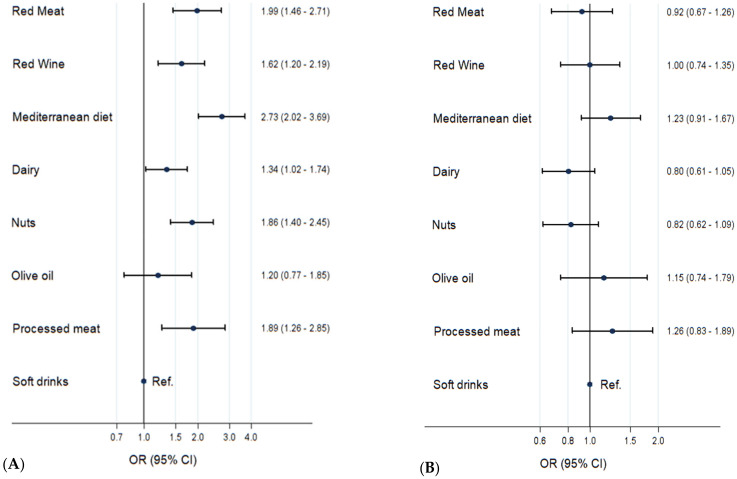
ORs and 95% CI for liking (**A**) and retweeting (**B**) a tweet related to the specific food groups of the study. The forest plots represent the different relative odds of a tweet sent by the selected US media outlets to be liked (**A**) and retweeted (**B**) compared to the soft drink group (reference category).

Tweets related to the MedDiet as a whole were the most liked tweets, having almost three times greater odds of being liked than the reference category, soft drinks (OR= 2.73, 95% CI: 2.02–3.69; Figure 3, first panel). Similarly, tweets about red meat, processed meat and nuts had nearly two times greater odds of being liked than the reference category of soft drinks tweets. By contrast, olive oil tweets had similar odds of being liked compared with soft drinks tweets (Figure 3).

By contrast, when we analyzed the probability of each food component of being retweeted, we found that none of them had greater probability of being retweeted than soft drinks (Figure 3, second panel).

Next, we calculated the probability of tweets on the MedDiet as a whole of being liked or retweeted as compared to those on dairy products, the food group with the highest number of tweets and retweets. We found that tweets about the MedDiet as a whole had significantly higher odds of being liked (OR = 2.04; 95% CI 1.72–2.43) and retweeted (OR = 1.54; 95% CI 1.29–1.83) than dairy tweets.

### 3.4. Number of Mass Media Tweets and Followers Retweets over Time (2009–2019)

**Figure 4:**
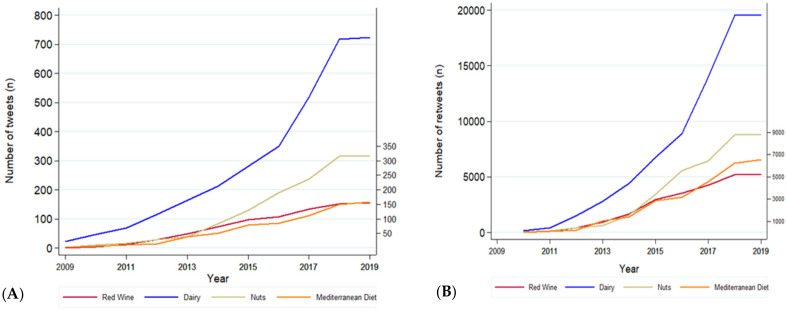
Time trend of all tweets (**A**) posted by the 25 major US media outlets selected in the study and the retweets generated by Twitter users (**B**) between 2009 and 2019. The data are shown for food groups that generated at least 15 tweets per year.

We assessed the evolution of the number of tweets related to the MedDiet or its component foods posted by the 25 US media outlets analyzed between 2009 and 2019. In parallel, we also studied the kinetics of the retweets that were generated (Figure 4). Throughout the analyzed years, we observed a progressive increase in the number of tweets and retweets generated on the MedDiet or its components by these media outlets. Nevertheless, this increase was not homogeneous across groups and during the years included in our observation. Dairy experienced a more pronounced increase in the number of tweets as years passed. On the other hand, it is worth noting that we observed a particular peak in the number of tweets related to nuts from 2014 to 2018 followed by a plateau. However, this trend observed in tweets related to nuts was not accompanied by a similar increase in the number of retweets. With regard to retweets, MedDiet experienced a remarkable peak from 2016 to 2018 followed by a steady growth.

**Figure 5:**
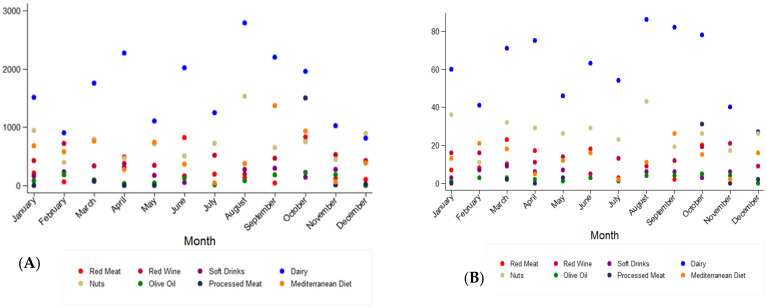
Monthly distribution of tweets (**A**) posted by 25 US media outlets and retweets (**B**) generated by Twitter users between 2009 and 2019. The scatter plot represents all the tweets (**A**) posted by the 25 major US media outlets selected in the study and the retweets (**B**) generated by their followers. Each food group is represented by a colored dot according to the following categories: red meat, red wine, soft drinks, dairy, nuts, olive oil, processed meat, and Mediterranean diet. Comparisons between months were not significant (figure (**A**): *p*-value = 0.91, figure (**B**): *p*-value = 0.73). *p*-value by Kruskall–Wallis H test.

In addition, we also investigated the number of tweets and retweets generated by month of the year. The Kruskal–Wallis H test showed that there were no statistically significant differences in the number of tweets (*p* = 0.91) or retweets (*p* = 0.73) between months (Figure 5).

**Figure 6:**
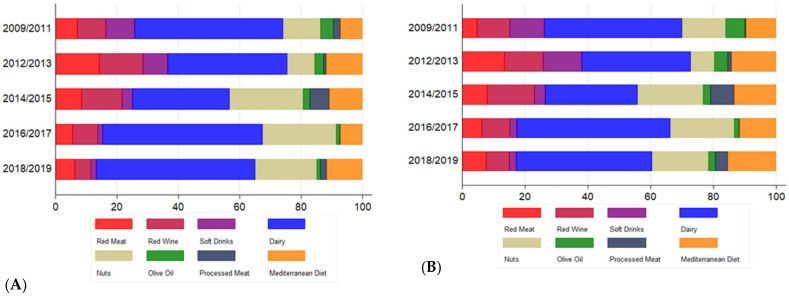
Proportion of tweets (**A**) and retweets (**B**) generated by each food group between 2009 and 2019. Initially aggregated into a three-year period, and afterwards into periods of two years. X-axis: Percentages (%) were calculated with respect to the total number of tweets and retweets, respectively.

We also examined the temporal distribution of the content along the analyzed period of time. The percentage of tweets with contents on dairy products was always the highest. Tweets referring to olive oil and soft drinks have progressively diminished whereas tweets related to nuts have progressively increased. Red meat maintains a similar proportion throughout the assessed time period. Tweets referring to processed meats clearly peaked in the years 2014 and 2015. Similarly, red wine-related tweets had a peak in the years 2012 and 2014 (Figure 6). Trends were similar in retweets, but some nuances are important to highlight. Olive oil experienced a mild increase in the percentage of retweets at the beginning of the study (2009–2011), while MedDiet experienced it at the end (2018–2019). On the other hand, soft drinks accumulated its higher percentage of retweets in the 2012–2013 period.

## 4. Discussion

### 4.1. Principal Findings

In this study, we have found that the main US media outlets were more interested in the single elements of the MedDiet than in the dietary pattern as a whole, with the greatest interest in dairy products. Interestingly, the attention given by the media to the different food groups has remained nearly stable over the last decade. Furthermore, Twitter users’ response in relation to the MedDiet and its elements was quantitatively related to the number of tweets posted by the US outlets in this regard, with the exception of those related to olive oil and MedDiet as a whole. Finally, none of the MedDiet components had a higher probability of being retweeted than the MedDiet itself.

Dietary habits play a critical role in the health of the individuals, and nutrition is considered a very relevant element for the prevention of chronic diseases and for health promotion [42,43]. Health research practice has changed dramatically over the past decade, largely due to methodological advancements [44]. Social media platforms such as Twitter are increasingly being leveraged by researchers for surveillance as well as to explore complex social issues such as perceptions of the public on chronic health conditions including diabetes, cardiovascular disease or obesity [45]. Furthermore, recognized social influential agents such as media outlets use Twitter as a dissemination tool for their information including health-related news [46]. When media outlets share information, their reach can be enormous. Thus, it is important that they may share contents which help to promote healthy habits in their Twitter accounts [47]. Social media has become ubiquitous, with more people accessing Web-based contents by following links on social media than through direct searches [48].

### 4.2. Communication Media and Diet

Our data show that the number of tweets sent by 25 major US outlets about the MedDiet or its elements has increased along the past decade despite the plateau observed last year. Interestingly, this increase was not homogeneous among groups, with dairy experiencing the greatest increase in number of tweets along the years of the study. Nonetheless, US outlets are overall more interested in physical and mental diseases than in the MedDiet as previously reported in a study that analyzed the tweets sent by 15 major US media outlets during one decade [46]. Different non-mutually exclusive reasons may explain this relatively poor interest of the major US outlets for the MedDiet. First, it may be in the best interest of business organizations such as pharmaceutical companies and health providers to focus on disease diagnosis and treatment rather than on promoting health and preventing disease [49,50]. Second, the limited scientific impact obtained by nutritional and epidemiological articles in comparison with those covering diagnosis and treatment of disease. Third, wide sectors of society potentially show a higher interest in knowledge on the diagnosis, treatment and prognosis of disease than in the promotion of healthy habits and primary disease prevention [51]. Fourth, some food companies (e.g., producers of dairy or nuts) may be especially active in promoting through social media the purported health benefits associated with consumption of their products and not so much in the more scientifically sound concept of high-quality food patterns [52,53]. In fact, food, beverage, and snack companies are increasingly promoting their brands on social media platforms, and utilize posts to advertise unhealthy products [54]. This fact may have contributed to our observed prominence of dairy products in social media, as compared to the relatively scarce media relevance of olive oil, despite the fact that olive oil is the hallmark of the MedDiet.

In our study, differences have been found in the number of tweets generated by each of the components included in the definition of the MedDiet. In particular, it is important to note that these differences have been augmenting during the years of our study. That is, the food group that gathered the most attention in the beginning of the decade (dairy) retained attention through the years, while other groups such as olive oil did not observe an increase in attention despite scientifically robust studies showing strong evidence of benefit for health [55]. Even more concerning is the fact that despite this strong evidence, we did not observe a correlation in the interest of the media on this topic [56,57]. Furthermore, the relative weight given to each component, as defined by the percentage of tweets received, was not related to the current scientific evidence of each component’s effects on health. The US media outlets showed their highest interest in dairy, even though dairy is a non-pivotal component of the MedDiet, and it is often negatively weighted with one point given if the consumption of dairy is below the median [58]. This finding could be explained by the fact that references to dairy are related to a wide range of milk-derived foods and beverages. On the other hand, other elements of the MedDiet, such as nuts, have been the object of interest for both US outlets and scientific studies that have reported, among others, the health benefits of nuts consumption [59,60]. Red meat (also opposed to the concept of MedDiet) and red wine (usually included in the definition of MedDiet) have also generated great interest in both the media and the medical literature [55]. However, controversy exists on the effects of wine or moderate alcohol consumption on health [61].

In contrast to the above-mentioned MedDiet components, olive oil has robust scientific evidence of its beneficial health properties such as preventing cardiovascular disease [55]. Yet, olive oil only accounted for a marginal percentage of the total tweets. This low interest of US media outlets may be reflective of the lower consumption of olive oil in the US in comparison with the Mediterranean countries [62]. In this regard, it is worth highlighting the interest that olive oil raised among Twitter users as opposed to the main US media outlets. One possible explanation could be that users coming from other countries other than the US where olive oil is better known such as Spain, Italy or Greece contributed to the higher number of tweets [63]. However, this is highly unlikely due to the fact that the tweets analyzed in this study were posted by US media outlets and were written in English. Thus, it is possible that the interest in the MedDiet by the Twitter community (i.e., general public) is higher than the interest shown by the main US outlets.

In addition, we have also analyzed the tone of the content of the tweets posted by the major US media outlets. Our data show that the media outlets analyzed employ a similar tone for all of the components of the MedDiet. Thus, a bias in the information related to this food pattern is not detected. Media outlets had a neutral attitude toward all the components of MedDiet, except for red wine. This may indicate that media outlets are not strongly concerned with any of the components analyzed. This may imply that they underestimate the key relevant role that diet plays in cardiovascular and chronic diseases, among many others [64].

Nonetheless, it is important to highlight that tweets about soft drinks were the least liked among Twitter users. Many studies have found that the consumption of soft drinks is unhealthy [65,66]. In fact, reducing soft drink consumption has been identified as one of the most important points of adherence to Mediterranean diet in the PREDIMED study [55]. This finding may reflect actual changes in social consideration toward soft drinks despite efforts made by some multinational soda corporations to undermine public health policies in this regard [67].

### 4.3. Media Persuasion

The important role of communication media outlets in generating popular opinion and emotions via information distribution has been clearly established in our society [68]. Furthermore, a recent study identified mass media as the main source for providing information on health research to the general public [1]. Thus, it is particularly worrying that our results suggest that the promotion of the health benefits of the MedDiet is not a relevant area of interest for US media outlets. As reported in 2015 by the National Health and Nutrition Examination Survey (NHANES), about three-fourths of the population in the US followed an unhealthy eating pattern [69]. This is of concern, given that poor diet quality is the primary cause of chronic disease and mortality in the US [70]. Adoption of the MedDiet is desirable for reversing this situation based on its health benefits, as shown in non-Mediterranean countries [41]. However, according to our results, media outlets in the US show insufficient awareness on these benefits, at least as far as the volume of tweets published is concerned. Assessing media outlet posts in Twitter can help understand the degree of awareness of the population with Mediterranean diet. It has been shown that there is an association between the characteristics of the content published in certain geographic areas and the rate of obesity and diabetes mellitus in that area [71]. In fact, a study showed that those areas with the most tweets about healthy foods had lower obesity rates [72]. The clinical management and treatment of disease attracts more attention from US media outlets than does the promotion of healthy habits [73].

Furthermore, many studies have found that mass media have a key role in persuasion, agenda setting, attitude formation, diffusion of knowledge and numerous other topics [74]. Indeed, media outlets should not be merely trying to influence policy but also try to educate and induce others to develop healthy behaviors and give voice to research. Thus, involvement of media outlets is desirable for promoting the MedDiet among Americans, as recommended in the 2015–2020 Dietary Guidelines [75]. Posting links to scientific articles can expand readership to a wider audience; for example, three tweets about a Cochrane review increased hits to its Webpage threefold, and readers linking to the Webpage via Twitter spent threefold more time on the page than those arriving from other sources [76]. Collective health behavior has a powerful impact on individual behavior [77].

### 4.4. Limitations

In order to interpret the results correctly some limitations should be addressed. The relevance of Twitter as a marker of social interest is a matter of controversy. Besides, the lack of data regarding the geographical location of Twitter users is a limitation when interpreting engagement. In addition, US news media outlets do not necessarily reflect the interests of society and might be influenced by financial conflicts of interest by companies that may use social media as a new way of marketing for their products. Large media outlets can also have a different set of priorities than news media in general. Regarding the collection of tweets, there is the risk that some were not detected since they may have used different hashtags. In contrast, our research tool has access to 100% of tweets, the strategy followed to define the hashtags and the period of time analyzed, that it’s the longest we have knowledge of, are among the strengths of our study [78]. Having public health and clinical practitioners carrying out the search minimizes possible bias on the choice of hashtags. Moreover, it can be argued that some categories included more hashtags than others. Finally, we decided to exclude those tweets that included hashtags referring to fruits, fish and vegetables because in our preliminary Twitter search prior to carrying out the study, we found that the content in most cases referred to sales, product advertising, harvesting or farming issues, restaurant menu offers, etc.

## 5. Conclusions

To our knowledge, this study is the first to assess the presence of MedDiet or its most relevant components in media outlets. US media outlets showed limited interest in the MedDiet, whereas the Twitter community showed a greater interest. Despite the fact that the MedDiet receives great scientific support as shown by the numerous studies reporting the positive effects this diet has on health, it is not the trendiest diet in the US. We believe that such a discrepancy may be influenced by a lack of diffusion of the positive effects of the MedDiet by the mass media. Understanding the public view of the MedDiet is necessary to design promotional strategies aimed at the appropriate population in order to increase adherence to MedDiet among the US population. Although this study is focused on US media, these results provide relevant information that probably can be applicable to other countries. Participation of researchers and health providers in related news and debates might create a collective awareness based on scientific data.

## Figures and Tables

**Table 1:** Number of tweets posted from 2009 to 2019 by 25 major US media outlets about the Mediterranean diet and the likes and retweets generated by Twitter users. The percentages (%) are calculated with respect to the total number of tweets.

	Tweets		Likes		Retweets	
	*n* (Frequency)	% (Percentage)	*n* (Frequency)	Number Likes/Number Tweets	*n* (Frequency)	Number Retweets/Number Tweets
Red meat	127	7.9	11,710	92.2	3951	31.1
Red wine	155	9.6	11,680	75.4	5231	33.8
Soft drinks	60	3.7	2786	46.4	2026	33.8
Dairy	723	45.0	44,877	62.1	19590	27.1
Nuts	317	19.7	27,327	86.2	8786	27.7
Olive oil	31	1.9	1721	55.5	1205	38.9
Processed meat	38	2.4	3337	87.8	1611	42.3
Mediterranean diet	157	9.8	19,925	126.9	6546	41.7
Total (or mean)	1608	100	123,363		48,946	

## Data Availability

The data that support the findings of this study are available from the corresponding author upon reasonable request.
